# Multivariate Analyses of Rotator Cuff Pathologies in Shoulder Disability

**DOI:** 10.1371/journal.pone.0118158

**Published:** 2015-02-24

**Authors:** Jan F. Henseler, Yotam Raz, Jochem Nagels, Erik W. van Zwet, Vered Raz, Rob G. H. H. Nelissen

**Affiliations:** 1 Department of Orthopaedics, Leiden University Medical Center, Postzone J-11-R, PO box 9600, 2300 RC Leiden, the Netherlands; 2 Department of Medical Statistics and Bioinformatics, Leiden University Medical Center, Leiden, the Netherlands; 3 Department of Human Genetics, Leiden University Medical Center, Leiden, the Netherlands; West Virginia University School of Medicine, UNITED STATES

## Abstract

**Background:**

Disability of the shoulder joint is often caused by a tear in the rotator cuff (RC) muscles. Four RC muscles coordinate shoulder movement and stability, among them the supraspinatus and infraspinatus muscle which are predominantly torn. The contribution of each RC muscle to tear pathology is not fully understood. We hypothesized that muscle atrophy and fatty infiltration, features of RC muscle degeneration, are predictive of superior humeral head translation and shoulder functional disability.

**Methods:**

Shoulder features, including RC muscle surface area and fatty infiltration, superior humeral translation and RC tear size were obtained from a consecutive series of Magnetic Resonance Imaging with arthrography (MRA). We investigated patients with superior (supraspinatus, n = 39) and posterosuperior (supraspinatus and infraspinatus, n = 30) RC tears, and patients with an intact RC (n = 52) as controls. The individual or combinatorial contribution of RC measures to superior humeral translation, as a sign of RC dysfunction, was investigated with univariate or multivariate models, respectively.

**Results:**

Using the univariate model the infraspinatus surface area and fatty infiltration in both the supraspinatus and infraspinatus had a significant contribution to RC dysfunction. With the multivariate model, however, the infraspinatus surface area only affected superior humeral translation (p<0.001) and discriminated between superior and posterosuperior tears. In contrast neither tear size nor fatty infiltration of the supraspinatus or infraspinatus contributed to superior humeral translation.

**Conclusion:**

Our study reveals that infraspinatus atrophy has the strongest contribution to RC tear pathologies. This suggests a pivotal role for the infraspinatus in preventing shoulder disability.

## Introduction

Shoulder complaints are the second largest cause for musculoskeletal disability in the middle aged and older populations. Shoulder complaints restrict daily functioning due to pain and limited arm mobility [1–3]. The majority of these complaints are caused by degenerative rotator cuff (RC) pathologies, ultimately leading to RC tears [3–6]. Degenerative RC pathology is the main cause of upper-limb related complaints in general practice [7,8], rheumatology [9] and orthopaedic clinics [10]. The prevalence of RC tears in the general population is high (20%) and increases with age (over 50% in the population above 65 years) [11–13].

The shoulder joint requires active stabilization from the RC muscles due to its non-constrained nature and dislocating forces of the prime shoulder movers (e.g. deltoids) [14–18]. In contrast to other joints like the hip, bony structures in the shoulder joint do not provide the same level of passive stability. The lack of passive stability allows positioning of the arm in space with an unsurpassed range of motion. The trade-off for mobility in the shoulder joint requires active compensatory stabilization by the RC muscles: the supraspinatus (SSp), infraspinatus (ISp), subscapularis and teres minor. In general, the SSp and ISp are more affected in RC tearing compared to the other two muscles [19]. The most frequent torn RC muscle is the SSp (i.e. superior tear), but a tear can progress posteriorly towards the ISp (i.e. posterosuperior tear). In RC tears the dynamic stabilization of the shoulder is lost. In the long term RC dysfunction can result in superior translation of the humeral head towards the acromion [14,16,20–24]. In large RC tears a decrease in acromiohumeral (AH) distance was reported [23,24]. In addition, fatty infiltration is also frequently found in RC muscle tear [21,25]. Both the AH distance and fatty infiltration are associated with poor surgical outcome and increased post-operative re-tear rates [26,27]. However, the intricate associations between superior translation of the humeral head (i.e. decrease in AH distance), RC muscle atrophy, fatty infiltration and RC tear size are not yet fully understood.

We aim to identify predictors of superior translation of the humeral head in the presence of superior and posterosuperior RC tears based on Magnetic Resonance imaging with arthrography (MRA). We hypothesized that features of the RC muscle (muscle atrophy and fatty infiltration) together with RC tear size will predict the degree of superior humeral head translation.

## Methods

### Patients

A retrospective study on all consecutive shoulder Magnetic Resonance imaging with arthrography (MRA) performed between January 2009 and December 2011 was undertaken. All patients were seen at the outpatient clinic of the department of Orthopaedics (Leiden University Medical Center, Leiden, the Netherlands) after complaints of shoulder pain and loss of range of motion and a clinical suspicion of a RC tear. The institutional ethic review board in Leiden (Committee Medical Ethics Leiden University Medical Center (CME)) approved the study. Since this is a retrospective study, the CME waived the need for written informed consent from the participants in this study.

**Fig 1:**
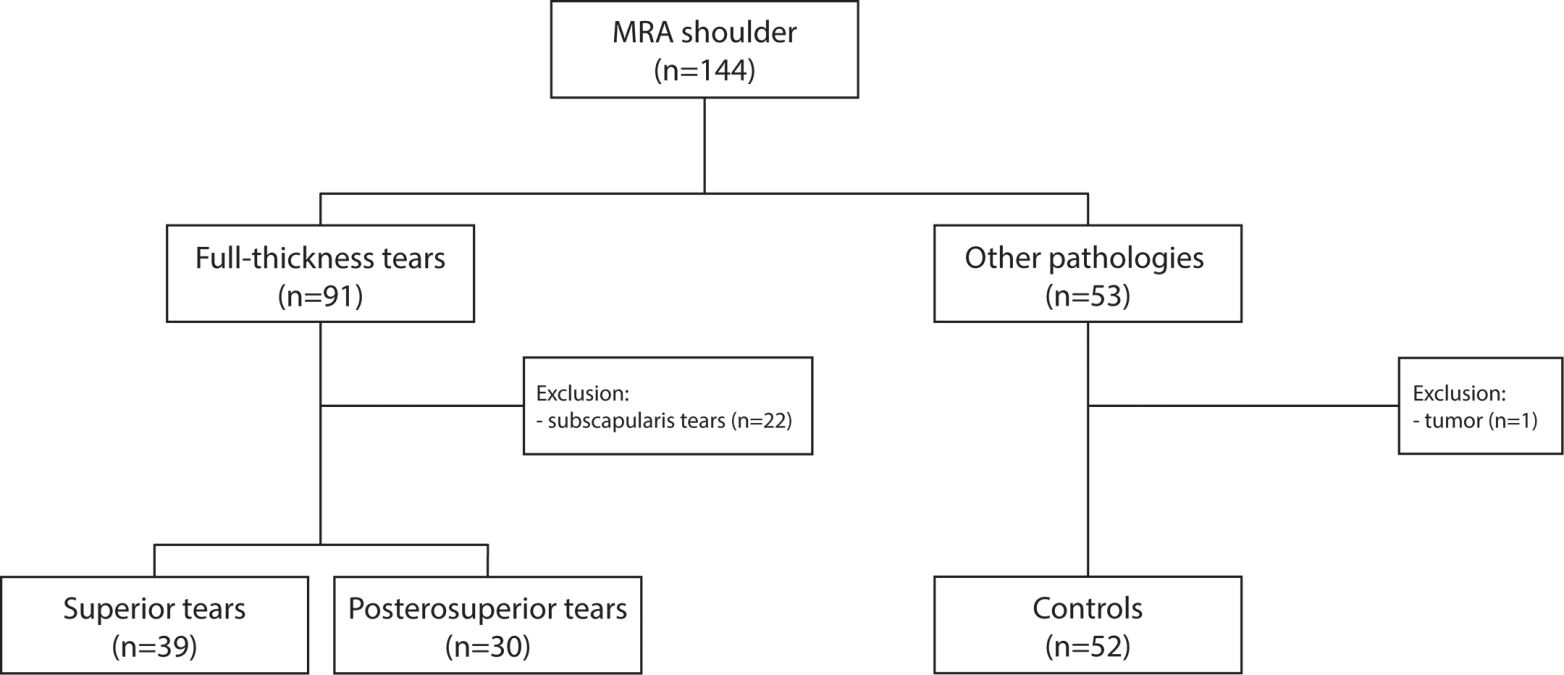
Flow chart of patient inclusion and exclusion.

Patient cases were selected through the hospital financial administration with the diagnosis of ‘rotator cuff tear’ (national Diagnosis Related Group code 1460) and ‘rotator cuff tendinitis’ (DRG code 1450). Exclusion criteria consisted of poor quality MRA scans (motion artifacts, poor view delineation), tearing of the subscapularis, deltoid and/or teres minor, history of fractures of the shoulder, tumors, severe shoulder joint deformity (glenohumeral osteoarthritis or rheumatoid arthritis grade III–IV), neurological denervation, stroke, muscular dystrophies, and myositis. A flow chart summarizing patient inclusion and exclusion is presented in Fig. 1. 144 patients with an MRA of the shoulder were identified and evaluated for inclusion. In total: 91 superior and posterosuperior RC tear patients were identified. 22 subscapularis tears and 1 tumor were excluded. 52 patients without a full-thickness RC tear were used as a control group. Of these, 8 patients had a partial thickness RC tear of the SSp, 8 patients were diagnosed with acromioclavicular (AC) osteoarthritis and 36 patients had unremarkable MRA scans.

### MRA Imaging procedure

Fifteen minutes before MRA, injection of contrast fluid into the glenohumeral joint was performed via a posterior approach under fluoroscopic guidance. Patients had local intra-articular anesthesia with 10mL of 1% lidocaine and subsequently a diluted Gd-DTPA mixture (i.e. 10cc NaCl, 5ml Marcaine 0.25% and 0.2ml Gadolinium 1:200) was injected. All MRA scans were performed on a 1.5 Tesla Avanto Siemens MRI unit (Siemens AG, Erlangen, Germany) or Philips Intera MRI unit (Philips Medical Systems, Best, the Netherlands) using a dedicated shoulder coil and turbo spin-echo sequences. Patients were scanned in a supine position with the arm in neutral rotation.

Analyses of the images were performed on a PACS Workstation with Sectra IDS5 (Sectra Medical Systems AB, Linköping, Sweden) as monitor readings. Image analysis was carried out by two independent observers who were blinded to the MRA report and diagnoses of the patients. As multiple planes and sequences were obtained following the institutional standard shoulder MRA protocol, the T1-weighted coronal and sagittal plane (TR/TE 500–600/11–15, matrix 256; slice thickness 4mm, inter-slice gap 1mm, field of view of 15cm) were systematically evaluated.

**Fig 2:**
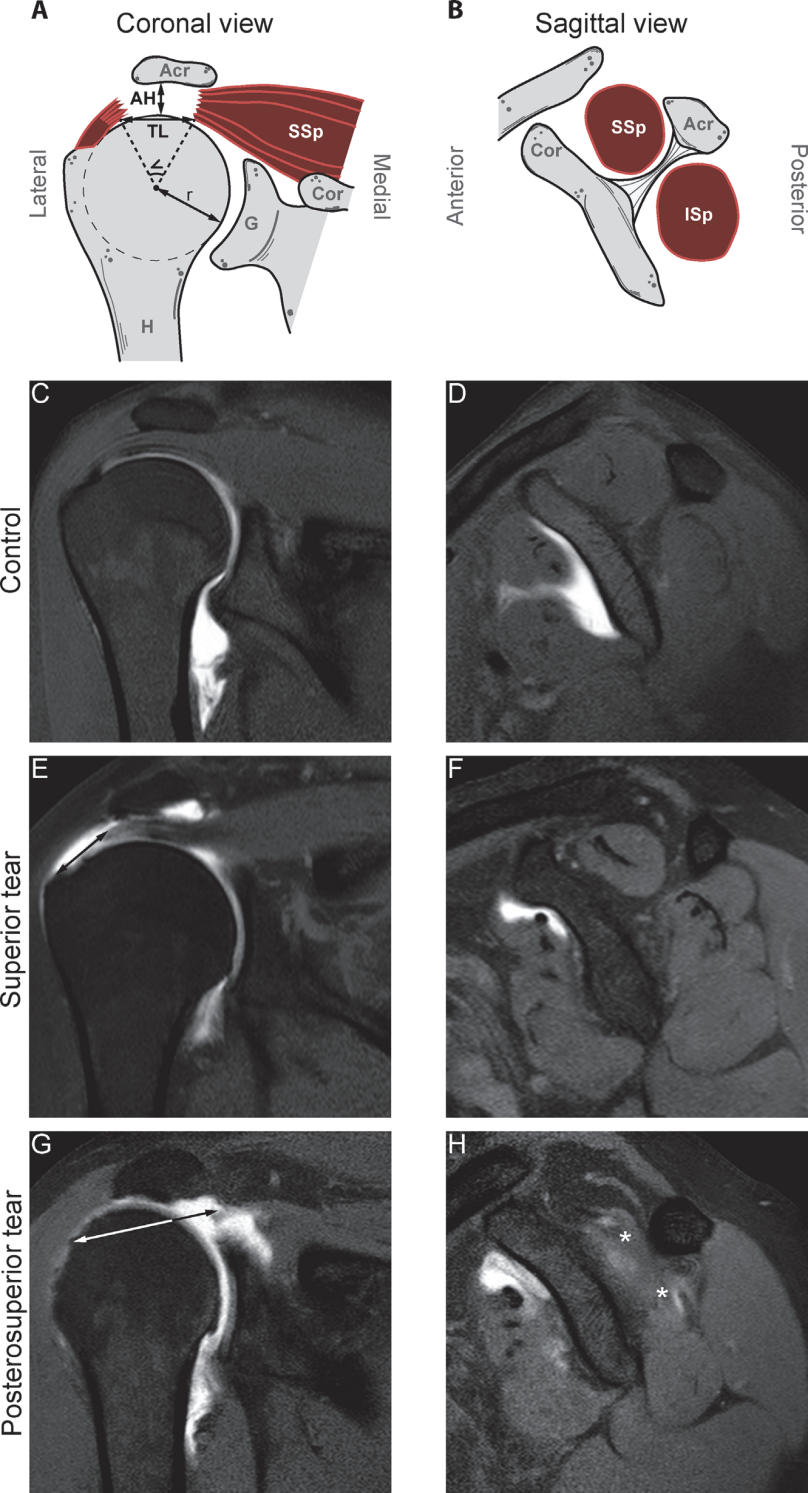
Schematic illustration of the measurements and representative MRA scans of coronal (A, C, E, G) and sagittal (B, D, F, H) of the shoulder in the patient groups. Anatomical landmarks are written in gray or white: acromion (Acr), supraspinatus (SSp), coracoid (Cor), glenoid (G) and humerus (H). Measurements are written in black: acromiohumeral distance (AH), tear length (TL), angle of tear (), radius of humerus (r), cross sectional surface area of the supraspinatus (SSp) and cross sectional surface area of the infraspinatus (ISp). For the control group, representative MRA images can be found in Fig. 2 panel C and D (coronal and sagittal view, respectively). For the superior tear group, representative MRA images can be found in Fig. 2 panel E and F (coronal and sagittal view, respectively). For the posterosuperior tear group, representative MRA images can be found in Fig. 2 panel G and H (coronal and sagittal view, respectively). In panels E and G the length of the rotator cuff tear is measured in the coronal plane (arrow). In panel H fat infiltration of the SSp and the ISp is indicated (*).

The methodology of the image evaluation is presented in Fig. 2A and 1B. The osteometry measurements performed on the coronal plane, at the widest point of the humeral head, are visualized in Fig. 2A [28]. The radius (r) of the humeral head was measured at its widest point using a circle-fit in the coronal plane. Superior translation of the humeral head was measured as the AH distance [24,29,30] and was defined as the smallest distance between the most cranial articular cortex of the humeral head, and the caudal cortical surface of the acromion determined in the coronal plane in the slice with the largest humeral head diameter (Fig. 2A). The radius of the humeral head and the AH distance are reported in mm.

Representative scans of the RC from a control are presented in Fig. 2C and 2D (coronal and sagittal view, respectively), for the superior tears in Fig. 2E and 2F (coronal and sagittal view, respectively) and for the posterosuperior tears Fig. 2G and 2H (coronal and sagittal view, respectively). In order to assess the anatomical features in a three-dimensional (3-D) perspective we measured the RC tear dimensions in the coronal and sagittal plane [31]. The tear geometry was measured on MRA as straight lines at the maximum anterior-to-posterior width in the coronal plane and maximum lateral-to-medial length in the sagittal plane (TL, Fig. 2A) as described previously [32]. The RC tear width in the coronal plane and length in the sagittal plane are reported in mm. To further approximate the RC tear size we measured of the RC tear size relative to the geometrical centre of the humeral head in the coronal and sagittal plane. The angle of the RC tear (, reported in degrees (°)) relative to the geometrical center of the humeral head was measured with the same anterior-to-posterior points on the coronal plane and the lateral-to-medial points of the tear in the sagittal plane (Fig. 2A). Subsequently, as the articular surface of the humeral head approximates a sphere, the RC tear surface area in cm^2^ could be calculated with the following formula:

$$\text{Tear surface area}=(\frac{{∠}_{coronal}}{360^{{}^{\circ}}}\times 2\pi \times {humerus}_{radius})\times (\frac{{∠}_{sagittal}}{360^{{}^{\circ}}}\times 2\pi \times {humerus}_{radius})$$

To determine muscular mass as a measure for muscle atrophy, the cross-sectional surface areas (CSA) of the SSp and the ISp were measured on the scan with the anatomical glenoid neck and base of the coracoid present, as illustrated in Fig. 2B. The CSA of the SSp and the ISp are reported in cm^2^. The presence of fatty infiltration was evaluated subjectively by examining either the presence or absence of intramuscular fatty infiltration in the SSp and ISp muscles on the coronal and sagittal T1-weighted images, as described previously with good inter-observer reliability [33,34].

### Statistical analyses

For the comparison of continuous variables between the patient groups one-way analysis of variance (ANOVA) with post-hoc testing were performed. For the comparison of categorical variables χ^2^ tests were performed.

The paired t-test was used to assess inter-observer differences. The Interclass correlation coefficient (ICC, two-way random model with absolute agreement) was used to assess the inter-observer reliability of the main continuous radiological variables. The Kappa (κ) was used to assess the inter-observer consistency of the nominal dichotomous parameters. For interpretation, the criteria formulated by Cicchetti and Sparrow were used [35]: ≤0.39, poor; 0.40 to 0.59, fair; 0.60 to 0.74, good; or ≥0.75, excellent.

We investigated whether muscle atrophy and fatty infiltration features of the RC had any effect on the AH distance. First, we considered all subjects and performed univariate analyses with the AH distance as numeric outcome, and SSp surface area (per cm^2^), ISp surface area (per cm^2^), SSp fatty infiltration (yes/no), ISp fatty infiltration (yes/no), age (years), gender and diagnosis (control/RC tear) as explanatory variables. Next, we performed a multivariate mixed model analysis with the AH distance as numeric outcome. The SSp and ISp surface area, SSp and ISp fatty infiltration, age, gender and diagnosis functioned all as explanatory variables into one multivariate model. The goal of such a multivariate model is to avoid confounding by assessing the effect of any single variable on the outcome, when all other variables are kept fixed. The categorical variables have a single effect and the continuous variables have an incremental effect. We performed similar analyses in the sub-population of RC tear patients. We therefore modified the explanatory variable diagnosis to indicate superior compared to posterosuperior tears. Also, we added tear size as an additional explanatory variable.

Statistical analyses were performed using SPSS Statistics (IBM Inc., Armonk, New York, USA). Statistical significance was considered with p-values of ≤0.05 (two-sided).

## Results

### Patient characteristics

**Fig 3:**
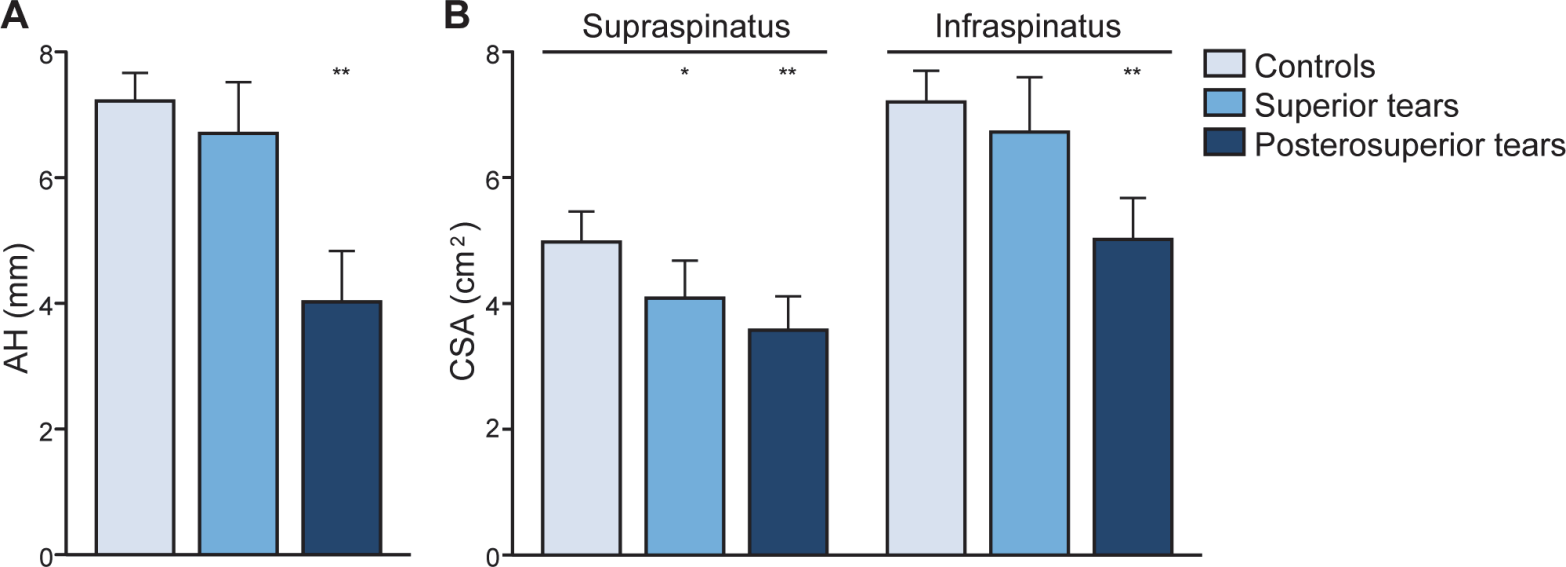
Means and standard errors of the means of the acromiohumeral distance and the cross sectional surface area of the supraspinatus and infraspinatus between the patient groups. Compared to controls: * p < 0.05; ** p < 0.001

Patient characteristics were stratified for diagnosis and are summarized in Table 1. Compared to controls, patients with a RC tear had a decreased AH distance (p = 0.02), and reduced surface area of the SSp and ISp muscles (p<0.001 and p = 0.002, respectively) (Fig. 3A). Patients with a RC tear were significantly older compared to controls (p<0.001). Patients with a RC tear had more fatty infiltration in SSp and ISp muscles (p<0.001 for both), compared to controls. Lastly, within the control group, the radius of the humeral head was strongly correlated with the surface of the SSp and ISp (Pearson correlation 0.566 and 0.350; p<0.001 and p = 0.04, respectively), whereas within the RC tear group only weak correlation was found between the radius of the humeral head and the surface of the SSp and ISp (Pearson correlation 0.203 and 0.120; p = 0.09 and p = 0.33, respectively) (Table 1).

**Table 1:** Patient Characteristics.

	Rotator cuff tears			
	Controls (N = 52)	Superior tears (N = 39)	Posterosuperior tears (N = 30)	p-value
Demographic data				
Age, years	46 (11.1)	58 (12.5)	62 (7.2)	<0.001
Female, N (%)	27 (51.9)	17 (43.6)	11 (36.7)	0.393
Left side, N (%)	28 (53.8)	17 (43.6)	6 (20.0)	0.011
Radiographic data				
AH distance, mm	7.2 (1.59)	6.7 (2.54)	4.0 (2.21)	<0.001
Radius of humeral head, mm	23.1 (1.93)	23.0 (2.06)	23.0 (1.7)	0.302
SSp surface, cm^2^	5.0 (1.73)	4.1 (1.87)	3.6 (1.47)	0.001
SSp fat infiltration, N (%)	8 (15.4)	13 (33.3)	20 (66.7)	<0.001
ISp surface, cm^2^	7.2 (1.77)	6.7 (2.72)	5.0 (1.80)	<0.001
ISp fat infiltration, N (%)	8 (15.4)	14 (35.9)	21 (70.0)	<0.001
Coronal tear length, mm	-	17 (10.3)	30 (13.5)	<0.001
Coronal tear angle, ˚	-	42 (26.4)	67 (30.8)	0.001
Sagittal tear length, mm	-	18 (11.3)	26.6 (12.9)	<0.001
Sagittal tear angle, ˚	-	44 (33.3)	71 (35.3)	0.002
Tear surface, cm^2^, median (IQR)	-	1.61 (0.80, 5.34)	7.41 (3.42, 14.21)	<0.001

The presented p-values are obtained through a one-way ANOVA. For the comparison of nominal variables χ^2^ tests were performed.

Next, we assessed whether these shoulder features could discriminate between patients with superior and posterosuperior tears. The AH distance was significantly reduced in patients with a posterosuperior tear (p = 0.002), as well as the ISp surface area (p = 0.007) (Fig. 3B). In addition, the length and angle of tear in the sagittal plane are larger (p = 0.01 and p = 0.046, respectively) and subsequently the RC tear size is also larger (p = 0.02) in patients with a posterosuperior tear. Furthermore, ISp fatty infiltration is more abundant in patients with posterosuperior tears (p<0.001).

### Inter-observer reliability and consistency

The inter-observer reliability are provided in Table 2. Every image was analyzed by two researchers independently. The AH distance measurements did not significantly differ between the two observers. Although small, there were statistical significant differences found for the ISp surface area and RC tear size. The ICC for the AH distance was 0.9 (p<0.001) and for the SSp and ISp surface areas 0.7 (p<0.001). This is considered good to excellent inter-observer reliability. For the dichotomous variables, the κ for the presence or absence (yes/no) of muscle fatty infiltration in either the SSp or ISp was 0.7 (p<0.001). This indicates good consistency between the two observers.

**Table 2:** Inter-observer difference and reliability.

	Interobserver difference			Reliabilty testing		
	Mean (SE)	95%-CI	p-value	ICC	95%-CI	p-value
AH distance, mm	0.2 (0.09)	-0.03–0.34	0.099	0.9	0.89–0.95	<0.001
Radius of humeral head, mm	0.4 (0.11)	0.17–0.63	0.001	0.8	0.70–0.86	<0.001
SSp surface, cm^2^	0.2 (0.13)	-0.54–0.48	0.117	0.7	0.63–0.81	<0.001
ISp surface, cm^2^	0.5 (0.17)	0.16–0.83	0.005	0.7	0.61–0.81	<0.001
Tear surface, cm^2^	1.2 (0.58)	0.12–2.38	0.031	0.8	0.63–0.84	<0.001

The inter-observer differences for the main continuous radiological features are obtained through paired t-tests. The ICC is obtained for reliability testing.

### The AH distance in RC tears and controls

The relation between the AH distance and muscle atrophy as the surface area (per cm2) and fatty infiltration (yes/no) features of the RC in controls and RC tear patients is summarized in Table 3.

**Table 3:** Contributors to acromiohumeral distance in RC tears and controls.

	Univariate models			Multivariate model		
Variable	Effect size	95%-CI	p-value	Effect size	95%-CI	p-value
Diagnosis (control) †	1.68	0.831–2.529	<0.001	0.76	-0.116–1.637	0.088
Surface area, cm^2^						
SSp	0.37	0.136–0.616	0.002	-0.07	-0.340–0.197	0.597
ISp	0.56	0.384–0.719	<0.001	0.52	0.304–0.725	<0.001
Fat infiltration (no) †						
SSp	2.38	1.540–3.211	<0.001	1.12	0.055–2.179	0.039
ISp	1.88	1.009–2.740	<0.001	0.04	-0.993–1.071	0.940
Age, yr	-0.05	-0.087–-0.020	0.002	0.01	-0.030–0.044	0.707
Gender (male) †	-0.39	-1.281–0.505	0.391	-0.77	-1.670–0.129	0.092

Univariate modelling evaluated the relation of each variable with the AH distance individually. Multivariate modeling evaluated the combined parameter estimates of the effect sizes of the variables on the AH distance in RC tears and controls.

† Categorical parameters have a single effect in the models, with fat infiltration (absence compared to presence), gender (male compared to female) and diagnosis (control compared to RC tear).

First the association of each RC feature as explanatory variable for the AH distance was assessed individually using univariate analyses. With the univariate model, the muscle surface area and fatty infiltration of both the SSp and the ISp muscle, age and diagnosis were associated with the AH distance (Table 3). The surface area of the SSp and ISp had an effect size of 0.37mm (p = 0.002) and 0.56mm (p<0.001) on the AH distance, respectively. This indicates an increase in AH distance of 0.37mm per 1cm2 SSp surface area or 0.56mm increase per 1cm2 ISp surface area. In the absence (no) of the SSp or ISp fatty infiltration there was an increase in AH distance of 2.38mm (p<0.001) or 1.88mm (p<0.001), respectively. Overall, the RC tear group had a 1.68mm smaller AH distance compared to the control group.

Next, we applied a multivariate model adjusted for age and gender to assess the contributions of the SSp and ISp surface areas and fatty infiltration on the AH distance between controls and RC tear patients (Table 3). In this model the ISp surface area remained influential indicating a significant contribution to the AH distance (0.5mm, p<0.001). Additionally, the absence (no) of fatty infiltration in the SSp was associated with an increase in AH distance (1.2mm, p = 0.039). In contrast, the SSp surface area, ISp fatty infiltration and the diagnosis (i.e. presence of an RC tear) did not significantly contribute to the AH distance.

### The AH distance in superior and posterosuperior tears

The associations of muscle atrophy and fatty infiltration features of the RC and the AH distance for superior and posterosuperior tears are summarized in Table 4. Using the univariate model, the contribution of ISp surface area, SSp and ISp fatty infiltration significantly to AH distance significantly differ between superior and posterosuperior tears (Table 4). In the univariate models, the ISp surface area had an effect size of 0.61mm per cm2 on the AH distance. The absence of fatty infiltration had a single effect of 2.29mm and 1.34mm for the SSp and ISp, respectively. The tear surface had a negative effect (-0.2mm, p<0.001), indicating a smaller AH in larger tears. Superior tears showed a larger AH distance compared to posterosuperior tears (2.68mm, p = 0.009).

**Table 4:** Contributors to acromiohumeral distance between superior and posterosuperior RC tears.

	Univariate models			Multivariate model		
Variable	Effect size	95%-CI	p-value	Effect size	95%-CI	p-value
Diagnosis (superior tear) †	2.68	1.512–3.846	<0.001	1.62	0.412–2.820	0.009
Surface area, cm^2^						
SSp	0.09	-0.075–0.261	0.272	-0.06	-0.485–0.354	0.755
ISp	0.61	0.383–0.828	<0.001	0.53	0.197–0.857	0.002
Fat infiltration (no) †						
SSp	2.29	1.087–3.495	<0.001	0.72	-0.972–2.408	0.399
ISp	1.34	0.049–2.620	0.042	-1.13	-2.527–0.273	0.113
Tear surface, cm^2^	-0.20	-0.288–-0.105	<0.001	-0.05	-0.162–0.51	0.304
Age, yr	-0.05	-0.109–0.014	0.131	-0.01	-0.060–0.057	0.690
Gender (male) †	0.05	-1.299–1.401	0.940	-0.33	-1.616–0.954	0.608

Univariate modelling evaluated the relation of each variable with the AH distance individually. Multivariate modeling evaluated the combined parameter estimates of the effect sizes of the variables on the AH distance in superior and posterosuperior RC tears.

† Categorical parameters have a single effect in the models, with fat infiltration (absence compared to presence), gender (male compared to female) and diagnosis (superior tear compared to posterosuperior tear).

With the multivariate model, however, only the ISp surface area remained associated with the AH distance (increase of 0.53 mm AH per 1cm^2^ ISp surface increase, Table 4). The model included the surface areas and fatty infiltration of the SSp and ISp and RC tear size with adjustments for age and gender. The superior tears had a larger AH distance compared to posterosuperior tears (1.62mm, p = 0.009, Table 4). We could not find that the tear surface contributed to the AH distance using the multivariate model, while a significant effect was found with the univariate model (Table 4).

## Discussion

Superior translation of the humeral head is a hallmark of advanced stage RC disease and leads to narrowing of the sub-acromial space with subsequent deterioration of shoulder function [14,16,20–24]. In the current study we found a decline in AH distance and increase in muscle atrophy and fatty infiltration in RC tears compared with controls. We found a similar decline in posterosuperior RC tears compared with superior RC tears. The ISp surface area most robustly differs between controls and RC tears and between posterosuperior and superior RC tears using a multivariate model.

In the current study we investigated the contributions of the SSp and ISp muscle surface area and fatty infiltration and tear size to AH distance. We found that the larger posterosuperior RC tears resulted in a smaller AH distance with more pronounced muscle atrophy and fatty infiltration compared to superior RC tears in agreement with previous studies [23,24]. However, in previous studies only individual RC features were assessed in relation to the AH distance, either RC tear size or fatty infiltration [21,23–25]. An association between the AH distance and RC tear size on ultrasound was found, but only for larger RC tear sizes [23]. Although we found a twofold increase in RC tear size for the posterosuperior tears compared to the superior tears, the contribution was less prominent compared to muscle atrophy and fatty infiltration. We are the first to apply an integrated model for multiple predictors to assess the AH distance in RC tears. With this model we identified the ISp surface area as the most important contributor to the AH distance. Therefore, we suggest the ISp surface area as a diagnosis predictor in RC disease.

The literature concerning the indication and timing of both surgical and conservative treatment for RC tears remains sparse. Still, pain scores and functional outcome results remain variable. Pain mediated shoulder adductor co-activation of the teres major and latissimus dorsi can compensate for lost RC function [18,36,37]. Although the glenohumeral stability can partially be restored with RC repair or tendon transfer surgery, similar gain is observed when strengthening the remaining RC and surrounding shoulder muscles [38–41]. This warrants further studies on the effect of pain medication such as corticosteroids treatments and physical therapy in order to exercise the remaining shoulder muscles and prevent them from degeneration. This ultimately will center the humeral head effectively onto the glenoid and retain shoulder functionality [14–18].

In the current study muscle surface area was measured on MRA of the shoulder. MRA provides a more accurate assessment of the RC tears in comparison to conventional MRI and ultrasound due to the contrast distending the joint capsule, outlining the intra-articular structures and dissemination into abnormalities [32,42,43]. Furthermore, several clinical score have been described to qualitatively assess RC features clinically. However, these classifications for RC muscle atrophy, fatty infiltration and tendon retraction did not reach satisfactory inter-observer reliability in previous studies [33,44]. We demonstrated that semi-quantitative measurements obtained with MRA of RC features are reliable, and subsequently could be used for statistical modeling. The relation between and the measurements of RC features used in the current study and these common shoulder classification systems used in the clinic will need further study.

Muscle atrophy and fatty infiltration are well known contributors to muscle degeneration in muscles dystrophies, myopathies, muscle aging and denervation [34,45]. Fatty infiltration is a non-specific response to local or systemic damage and considered as a common outcome in muscle degeneration, including muscular atrophy. However the causality of fatty infiltration in muscle degeneration pathogenesis is not fully resolved. Goutallier et al. reported that fatty infiltration in the RC muscles only occurs in presence of a RC tear [46]. However, Ashry et al. and others found progression of fatty infiltration in shoulder muscles with aging in patients without RC tears [34,47]. We confirmed an association between RC tear and fatty infiltration, although fatty infiltration was not found in all RC tears. We observed fatty infiltration also in the control group without RC tears. This suggests that fatty infiltration may only be indirectly associated with RC tears. Furthermore, it supports the idea that muscle atrophy and fat infiltration are independently associated processes [48].

This study has some limitations. The study is cross-sectional therefore the progression from superior to posterosuperior tears cannot be assessed. Furthermore, the control group does not consist of unaffected shoulders without symptoms, and are younger compared to the RC tear patients. Therefore the differences between RC tears and control groups could be under-estimated. Fatty infiltration was measured qualitatively, and accurate measurements of fatty infiltration should be quantitative. However excellent correlations were reported between previous quantitative measurements and a qualitative visual rating, as used in the current study [21]. Moreover, ideally longitudinal studies should unravel the rate and mechanism of progression from superior to posterosuperior tears and the progress of fatty infiltration of muscles towards the development of RC tears.

Despite the limitation of the current study, we demonstrated an association between the AH distance and RC muscle’s fatty infiltration and muscle atrophy. The AH distance, which reflects glenohumeral stability as a sign of RC dysfunction, is mostly affected by the remaining infraspinatus size, whereas the RC tear size has a lesser effect. This indicates a pivotal role for the infraspinatus within the RC muscles in preventing excessive superior translation of the humeral head.

## References

[1] Nakajima D, Yamamoto A, Kobayashi T, Osawa T, Shitara H, et al (2012) The effects of rotator cuff tears, including shoulders without pain, on activities of daily living in the general population. J Orthop Sci 17: 136–140. 10.1007/s00776-011-0186-4 22249436

[2] Roquelaure Y, Ha C, Leclerc A, Touranchet A, Sauteron M, et al (2006) Epidemiologic surveillance of upper-extremity musculoskeletal disorders in the working population. Arthritis Rheum 55: 765–778. 10.1002/art.22222 17013824

[3] Greving K, Dorrestijn O, Winters JC, Groenhof F, van der Meer K, et al (2012) Incidence, prevalence, and consultation rates of shoulder complaints in general practice. Scand J Rheumatol 41: 150–155. 10.3109/03009742.2011.605390 21936616

[4] Oh LS, Wolf BR, Hall MP, Levy BA, Marx RG. (2007) Indications for rotator cuff repair: a systematic review. Clin Orthop Relat Res 455: 52–63. 10.1097/BLO.0b013e31802fc175 17179786

[5] Dorrestijn O, Greving K, van der Veen WJ, van der Meer K, Diercks RL, et al (2011) Patients with shoulder complaints in general practice: consumption of medical care. Rheumatology (Oxford) 50: 389–395. 10.1093/rheumatology/keq333 21047806

[6] Matsen FA III. (2008) Clinical practice. Rotator-cuff failure. N Engl J Med 358: 2138–2147. 10.1056/NEJMcp0800814 18480206

[7] Bot SD, van der Waal JM, Terwee CB, van der Windt DA, Schellevis FG, et al (2005) Incidence and prevalence of complaints of the neck and upper extremity in general practice. Ann Rheum Dis 64: 118–123. 10.1136/ard.2003.019349 15608309 PMC1755209

[8] van der Windt DA, Koes BW, de Jong BA, Bouter LM. (1995) Shoulder disorders in general practice: incidence, patient characteristics, and management. Ann Rheum Dis 54: 959–964 8546527 10.1136/ard.54.12.959 PMC1010060

[9] Vecchio P, Kavanagh R, Hazleman BL, King RH. (1995) Shoulder pain in a community-based rheumatology clinic. Br J Rheumatol 34: 440–442 7788173 10.1093/rheumatology/34.5.440

[10] Dunn WR, Schackman BR, Walsh C, Lyman S, Jones EC, et al (2005) Variation in orthopaedic surgeons' perceptions about the indications for rotator cuff surgery. J Bone Joint Surg Am 87: 1978–1984. 10.2106/JBJS.D.02944 16140812

[11] Sher JS, Uribe JW, Posada A, Murphy BJ, Zlatkin MB. (1995) Abnormal findings on magnetic resonance images of asymptomatic shoulders. J Bone Joint Surg Am 77: 10–15 7822341 10.2106/00004623-199501000-00002

[12] Moosmayer S, Tariq R, Stiris MG, Smith HJ. (2010) MRI of symptomatic and asymptomatic full-thickness rotator cuff tears. A comparison of findings in 100 subjects. Acta Orthop 81: 361–366. 10.3109/17453674.2010.483993 20450423 PMC2876840

[13] Mall NA, Kim HM, Keener JD, Steger-May K, Teefey SA, et al (2010) Symptomatic progression of asymptomatic rotator cuff tears: a prospective study of clinical and sonographic variables. J Bone Joint Surg Am 92: 2623–2633. 10.2106/JBJS.I.00506 21084574 PMC2970889

[14] Steenbrink F, de Groot JH, Veeger HE, van der Helm FC, Rozing PM. (2009) Glenohumeral stability in simulated rotator cuff tears. J Biomech 42: 1740–1745. 10.1016/j.jbiomech.2009.04.011 19450803

[15] Hansen ML, Otis JC, Johnson JS, Cordasco FA, Craig EV, et al (2008) Biomechanics of massive rotator cuff tears: implications for treatment. J Bone Joint Surg Am 90: 316–325. 10.2106/JBJS.F.00880 18245591

[16] McCully SP, Suprak DN, Kosek P, Karduna AR. (2006) Suprascapular nerve block disrupts the normal pattern of scapular kinematics. Clin Biomech (Bristol, Avon) 21: 545–553. 10.1016/j.clinbiomech.2006.02.001 16603286

[17] Lugo R, Kung P, Ma CB. (2008) Shoulder biomechanics. Eur J Radiol 68: 16–24. 10.1016/j.ejrad.2008.02.051 18511227

[18] Steenbrink F, Meskers CG, Nelissen RG, de Groot JH. (2010) The relation between increased deltoid activation and adductor muscle activation due to glenohumeral cuff tears. J Biomech 43: 2049–2054. 10.1016/j.jbiomech.2010.04.012 20452596

[19] Melis B, Nemoz C, Walch G. (2009) Muscle fatty infiltration in rotator cuff tears: descriptive analysis of 1688 cases. Orthop Traumatol Surg Res 95: 319–324. 10.1016/j.otsr.2009.05.001 19586809

[20] Lehtinen JT, Belt EA, Kauppi MJ, Kaarela K, Kuusela PP, et al (2001) Bone destruction, upward migration, and medialisation of rheumatoid shoulder: a 15 year follow up study. Ann Rheum Dis 60: 322–326 11247859 10.1136/ard.60.4.322 PMC1753606

[21] van de Sande MA, Stoel BC, Obermann WR, Lieng JG, Rozing PM. (2005) Quantitative assessment of fatty degeneration in rotator cuff muscles determined with computed tomography. Invest Radiol 40: 313–319 15829828 10.1097/01.rli.0000160014.16577.86

[22] Hirooka A, Wakitani S, Yoneda M, Ochi T. (1996) Shoulder destruction in rheumatoid arthritis. Classification and prognostic signs in 83 patients followed 5–23 years. Acta Orthop Scand 67: 258–263 8686464 10.3109/17453679608994684

[23] Keener JD, Wei AS, Kim HM, Steger-May K, Yamaguchi K. (2009) Proximal humeral migration in shoulders with symptomatic and asymptomatic rotator cuff tears. J Bone Joint Surg Am 91: 1405–1413. 10.2106/JBJS.H.00854 19487518 PMC2686133

[24] Henseler JF, de Witte PB, de Groot JH, van Zwet EW, Nelissen RG, et al (2013) Cranial translation of the humeral head on radiographs in rotator cuff tear patients: the modified active abduction view. Med Biol Eng Comput. 10.1007/s11517-013-1057-2 23543305

[25] Fuchs B, Weishaupt D, Zanetti M, Hodler J, Gerber C. (1999) Fatty degeneration of the muscles of the rotator cuff: assessment by computed tomography versus magnetic resonance imaging. J Shoulder Elbow Surg 8: 599–605 10633896 10.1016/s1058-2746(99)90097-6

[26] Meyer DC, Wieser K, Farshad M, Gerber C. (2012) Retraction of supraspinatus muscle and tendon as predictors of success of rotator cuff repair. Am J Sports Med 40: 2242–2247. 10.1177/0363546512457587 22926748

[27] Kim JR, Cho YS, Ryu KJ, Kim JH. (2012) Clinical and radiographic outcomes after arthroscopic repair of massive rotator cuff tears using a suture bridge technique: assessment of repair integrity on magnetic resonance imaging. Am J Sports Med 40: 786–793. 10.1177/0363546511434546 22307079

[28] Rozing PM, Obermann WR. (1999) Osteometry of the glenohumeral joint. J Shoulder Elbow Surg 8: 438–442 10543596 10.1016/s1058-2746(99)90073-3

[29] Nagels J, Verweij J, Stokdijk M, Rozing PM. (2008) Reliability of proximal migration measurements in shoulder arthroplasty. J Shoulder Elbow Surg 17: 241–247. 10.1016/j.jse.2007.07.011 18234527

[30] van de Sande MA, Rozing PM. (2006) Proximal migration can be measured accurately on standardized anteroposterior shoulder radiographs. Clin Orthop Relat Res 443: 260–265. 10.1097/01.blo.0000196043.34789.73 16462449

[31] Davidson JF, Burkhart SS, Richards DP, Campbell SE. (2005) Use of preoperative magnetic resonance imaging to predict rotator cuff tear pattern and method of repair. Arthroscopy 21: 1428 10.1016/j.arthro.2005.09.015 16376230

[32] van der Zwaal P, Thomassen BJ, Urlings TA, de Rooy TP, Swen JW, et al (2012) Preoperative agreement on the geometric classification and 2-dimensional measurement of rotator cuff tears based on magnetic resonance arthrography. Arthroscopy 28: 1329–1336. 10.1016/j.arthro.2012.04.054 22885159

[33] Slabaugh MA, Friel NA, Karas V, Romeo AA, Verma NN, et al (2012) Interobserver and intraobserver reliability of the Goutallier classification using magnetic resonance imaging: proposal of a simplified classification system to increase reliability. Am J Sports Med 40: 1728–1734. 10.1177/0363546512452714 22753846

[34] Ashry R, Schweitzer ME, Cunningham P, Cohen J, Babb J, et al (2007) Muscle atrophy as a consequence of rotator cuff tears: should we compare the muscles of the rotator cuff with those of the deltoid? Skeletal Radiol 36: 841–845. 10.1007/s00256-007-0307-5 17508210

[35] Cicchetti DV, Sparrow SA. (1981) Developing criteria for establishing interrater reliability of specific items: applications to assessment of adaptive behavior. Am J Ment Defic 86: 127–137 7315877

[36] Henseler JF, Nagels J, Nelissen RG, de Groot JH. (2014) Does the latissimus dorsi tendon transfer for massive rotator cuff tears remain active postoperatively and restore active external rotation? J Shoulder Elbow Surg 23: 553–560. 10.1016/j.jse.2013.07.055 24135419

[37] Steenbrink F, de Groot JH, Veeger HE, Meskers CG, van de Sande MA, et al (2006) Pathological muscle activation patterns in patients with massive rotator cuff tears, with and without subacromial anaesthetics. Man Ther 11: 231–237. 10.1016/j.math.2006.07.004 16890886

[38] Gerber C, Maquieira G, Espinosa N. (2006) Latissimus dorsi transfer for the treatment of irreparable rotator cuff tears. J Bone Joint Surg Am 88: 113–120. 10.2106/JBJS.E.00282 16391256

[39] Henseler JF, Nagels J, van der Zwaal P, Nelissen RGHH. (2013) Teres major tendon transfer for patients with massive irreparable posterosuperior rotator cuff tears: Short-term clinical results. Bone Joint J 95-B: 523–529. 10.1302/0301-620X.95B4.30390 23539705

[40] Ainsworth R, Lewis JS. (2007) Exercise therapy for the conservative management of full thickness tears of the rotator cuff: a systematic review. Br J Sports Med 41: 200–210. 10.1136/bjsm.2006.032524 17264144 PMC2658945

[41] Moosmayer S, Lund G, Seljom US, Haldorsen B, Svege IC, et al (2014) Tendon repair compared with physiotherapy in the treatment of rotator cuff tears: a randomized controlled study in 103 cases with a five-year follow-up. J Bone Joint Surg Am 96: 1504–1514. 10.2106/JBJS.M.01393 25232074

[42] de Jesus JO, Parker L, Frangos AJ, Nazarian LN. (2009) Accuracy of MRI, MR arthrography, and ultrasound in the diagnosis of rotator cuff tears: a meta-analysis. AJR Am J Roentgenol 192: 1701–1707. 10.2214/AJR.08.1241 19457838

[43] Steinbach LS, Palmer WE, Schweitzer ME. (2002) Special focus session. MR arthrography. Radiographics 22: 1223–1246 12235350 10.1148/radiographics.22.5.g02se301223

[44] Lippe J, Spang JT, Leger RR, Arciero RA, Mazzocca AD, et al (2012) Inter-rater agreement of the Goutallier, Patte, and Warner classification scores using preoperative magnetic resonance imaging in patients with rotator cuff tears. Arthroscopy 28: 154–159. 10.1016/j.arthro.2011.07.016 22019235

[45] Klatte-Schulz F, Pauly S, Scheibel M, Greiner S, Gerhardt C, et al (2012) Influence of age on the cell biological characteristics and the stimulation potential of male human tenocyte-like cells. Eur Cell Mater 24: 74–89 22791374 10.22203/ecm.v024a06

[46] Goutallier D, Postel JM, Bernageau J, Lavau L, Voisin MC. (1994) Fatty muscle degeneration in cuff ruptures. Pre- and postoperative evaluation by CT scan. Clin Orthop Relat Res: 78–83 8020238

[47] Melis B, Wall B, Walch G. (2010) Natural history of infraspinatus fatty infiltration in rotator cuff tears. J Shoulder Elbow Surg 19: 757–763. 10.1016/j.jse.2009.12.002 20363160

[48] Barry JJ, Lansdown DA, Cheung S, Feeley BT, Ma CB. (2013) The relationship between tear severity, fatty infiltration, and muscle atrophy in the supraspinatus. J Shoulder Elbow Surg 22: 18–25. 10.1016/j.jse.2011.12.014 22541866

